# Preclinical Proof-of-Concept of a Minimally Invasive Direct Cardiac Compression Device for Pediatric Heart Support

**DOI:** 10.1007/s13239-023-00703-0

**Published:** 2023-12-18

**Authors:** Erica C. Hord, Melanie P. Hager, Christina M. Bolch, Katherine Bonugli, Lee-Jae Guo, Egemen Tuzun, John C. Criscione

**Affiliations:** 1 CorInnova, Inc. JLABS @ TMC, 2450 Holcombe Blvd Suite J, Houston, TX 77021 USA; 2 Department of Biomedical Engineering, Texas A&M University, 5045 Emerging Technologies Building 3120 TAMU, College Station, TX 77843-3120 USA; 3 Texas A&M University College of Medicine, 3050 Health Professions Education Building 1359 TAMU, Bryan, TX 77807-1359 USA; 4 Texas A&M University Institute for Preclinical Studies, 4478 TAMU, College Station, TX 77843-4478 USA

**Keywords:** Pediatric Heart Failure, Mechanical circulatory support, MCS, DCC, Medical Device

## Abstract

**Purpose:**

For pediatric patients, extracorporeal membrane oxygenation (ECMO) remains the predominant mechanical circulatory support (MCS) modality for heart failure (HF) although survival to discharge rates remain between 50 and 60% for these patients. The device-blood interface and disruption of physiologic hemodynamics are significant contributors to poor outcomes.

**Methods:**

In this study, we evaluate the preclinical feasibility of a minimally invasive, non-blood-contacting pediatric DCC prototype for temporary MCS. Proof-of-concept is demonstrated in vivo in an animal model of HF. Hemodynamic pressures and flows were examined.

**Results:**

Minimally invasive deployment on the beating heart was successful without cardiopulmonary bypass or anticoagulation. During HF, device operation resulted in an immediate 43% increase in cardiac output while maintaining pulsatile hemodynamics. Compared to the pre-HF baseline, the device recovered up to 95% of ventricular stroke volume. At the conclusion of the study, the device was easily removed from the beating heart.

**Conclusions:**

This preclinical proof-of-concept study demonstrated the feasibility of a DCC device on a pediatric scale that is minimally invasive and non-blood contacting, with promising hemodynamic support and durability for the initial intended duration of use. The ability of DCC to maintain pulsatile MCS without blood contact represents an opportunity to mitigate the mortality and morbidity observed in non-pulsatile, blood-contacting MCS.

**Supplementary Information:**

The online version contains supplementary material available at 10.1007/s13239-023-00703-0.

## Introduction

In the USA, the number of pediatric patients with heart failure (HF) refractory to medical management is expanding. For these patients, treatment requires orthotopic heart transplantation (OHT), surgical repair, and/or mechanical circulatory support (MCS). Notably, the active waitlist for pediatric OHT has grown by > 20% since 2010 [1]; however, the volume of pediatric OHT has not increased during this time period, leading to a growing number of pediatric patients dependent on MCS [2, 3].

Ventricular assist devices (VADs) and extracorporeal membrane oxygenation (ECMO) are the two most commonly utilized modalities for providing MCS to HF patients. Both of these modalities are blood-contacting. For pediatric patients, ECMO remains the preferred [4] and most frequently employed modality of MCS [5]. The goal is to temporarily provide circulatory support to the other organs until the heart can recover. In addition to the growing utilization of ECMO throughout pediatrics [6], the volume of operative repair of congenital heart disease (CHD) has also increased steadily over recent decades [7, 8], with an estimated 40,000 pediatric cardiac surgeries annually in the USA [9]. From 2000 to 2010, an estimated 2.4% of pediatric cardiac surgery cases required post-cardiotomy ECMO (PC-ECMO) [10]. The probability of requiring PC-ECMO is correlated with the complexity and severity of the lesion repaired; as surgeons continue to expand the range of treatable defects into increasingly complex pathologies, the use of PC-ECMO is expected to continue increasing [5]. Survival rates to discharge continue to be < 50% for patients supported by PC-ECMO [10, 11].

Several factors contribute to the 50% mortality observed with PC-ECMO. Complications arise from inflammatory response to foreign-body contact with blood and invasive vascular cannulation, including pump thrombosis, hemolysis, thromboembolism, infection, stroke, limb ischemia or amputation, and adverse neurological events [5]. Most importantly, anticoagulation regimens are required to limit thromboembolism, and yet anticoagulants are difficult to manage and can contribute to uncontrolled hemorrhage.

In addition to the numerous physiologic risks of using PC-ECMO, discontinuation of PC-ECMO, a temporary therapy, poses logistic and clinical management challenges. To evaluate a patient’s condition for discontinuing ECMO, a weaning trial is performed where the flow rate of the ECMO circuit is incrementally reduced while the patient is closely monitored. It is challenging to wean pediatric patients, as reducing the pump flow rate also increases the risk of pump thrombosis. Moreover, without the ability to fully suspend the support, it is difficult to determine if the patient is truly ready for MCS removal.

Improvements in cardiovascular support for pediatric patients may be realized by a device that is non-blood contacting and non-obligatory. Prior preclinical studies have demonstrated direct cardiac compression (DCC) devices are capable of providing non-blood contacting and non-obligatory MCS and have been recently summarized in review [12–14], however pediatric DCC work has been limited [15, 16]. In this paper, we describe a non-blood-contacting, minimally invasive, DCC prototype developed for pediatric-sized hearts. The prototype pediatric heart support system provides pulsatile support to the ventricles without blood contact by use of an intrapericardial DCC device controlled by a custom extracorporeal pneumatic driver. This method of heart support has the potential to mitigate current MCS pump-related complications that often lead to catastrophic outcomes. Furthermore, this technology is non-obligatory, meaning it can be turned off for extended periods without complication, thus allowing complete weaning. The prototype is collapsible and can be explanted through the transcutaneous driveline track. In this study, we demonstrated the proof-of-concept of this novel DCC implantation and design for pediatric heart support using an esmolol-induced acute HF caprine model.

## Materials and Methods

A single, healthy 25.5 kg Spanish caprine (goat) was used as the animal model for this study. The animal was cared for and assessed according to principles outlined in the National Research Council’s Guide for the Care and Use of Laboratory Animals. The study was approved by the Institutional Animal Care and Use Committee at Texas A&M University.

### Pre-Study Imaging for Heart Size and Prototype Development

**Fig. 1:**
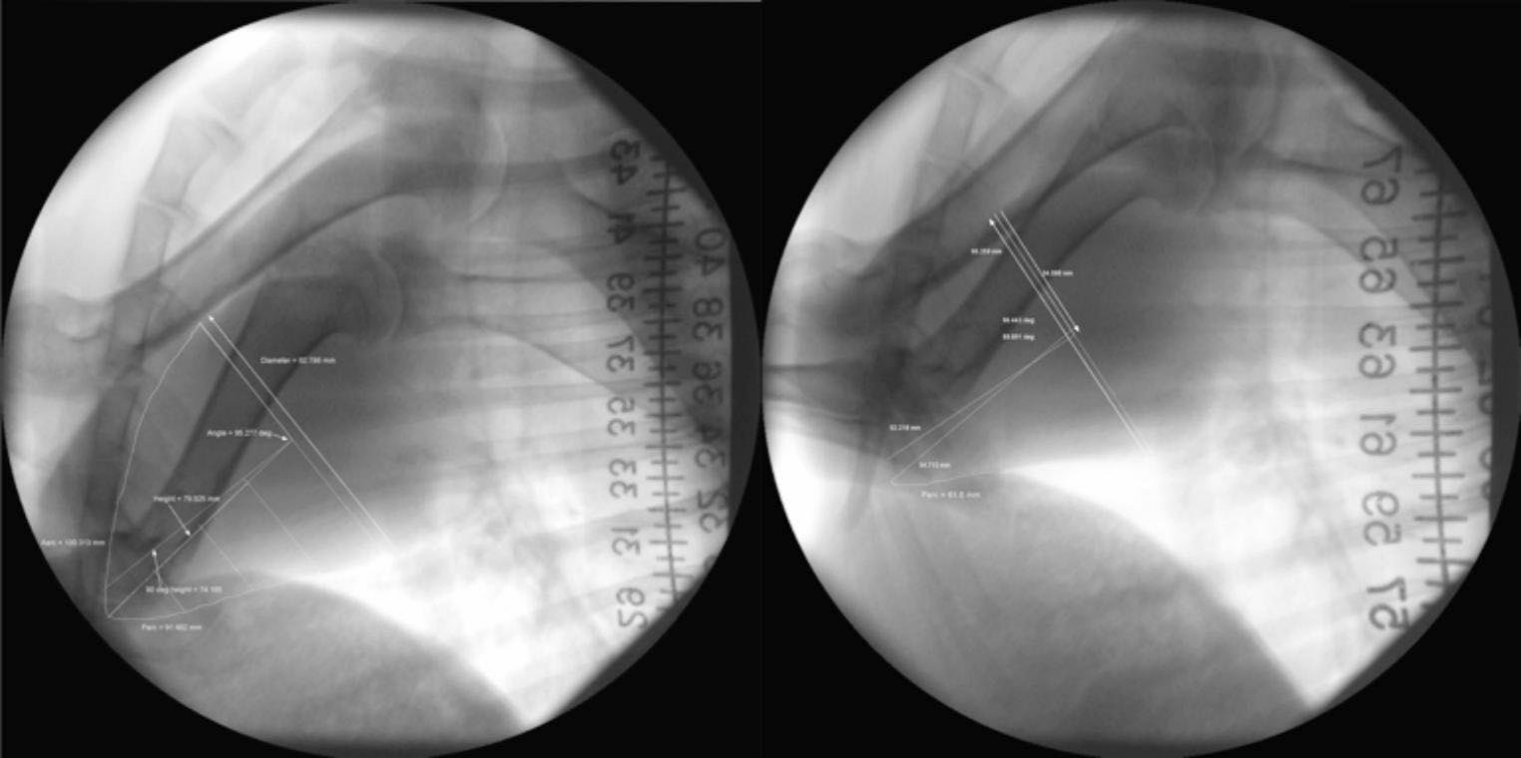
Fluoroscopic cine end-diastolic frames annotated with heart dimension measurements collected prior to the study; note the different sizes for the ruler scale when on the left and right of the animal (proximal and distal to the C-arm imaging intensifier)

Approximately 1 month prior to the implantation procedure, fluoroscopic imaging was performed in the conscious animal to measure the size of the heart and construct a custom size device. With the animal conscious and standing, the fluoroscopy C-Arm was positioned to obtain sagittal views of the chest, with the heart captured inside the images. Two cine loops were collected: one with a radiopaque ruler scale on the left side of the animal and one with the scale on the right. In both, the scale was positioned near the shoulder of the animal in the field of view (Fig. 1). Note that the scale is different in the images whereas the anatomy is similar. The average calibration from the two scales provided a dimension in the midline of the chest, the location of the heart in ruminants. A frame during end-diastole from each view was exported to define the target exterior dimensions of the heart, including the diameter at the base and apex-to-base length. Additional circumferential heart dimensions were measured between the apex and the base to generate a CAD drawing to model a symmetric version of the heart. This CAD model was then used for constructing the pediatric DCC implantable prototypes similar in design to the previously described CorInnova adult MCS design [17, 18]. In essence, the DCC implantable features a circumferential network of polyurethane bladders, a nitinol frame that facilitates self-expanding deployment from a small tube, and a smooth polyurethane outer containment that interfaces with the heart. The basal diameter of the pediatric implantable was 78 mm, which was compressed to 16 mm for deployment. However, the challenge with implantation in a pediatric size animal was more than just resizing the geometry of the device to fit a heart with a 25% reduction in diameter from a typical adult-sized preclinical heart model. Rather, a critical refinement was needed to reduce the device bulk enough to be compressed and deployed through a tube that was 16 mm in diameter (the adult sized device is deployed through a tube with 25 mm diameter). Basically, the bulk needed to be reduced by a much larger fraction (i.e. 60%) than the deployed geometry (i.e. 25%). Toward reducing bulk, a continuous hoop filament made of polyester was attached to the wire frame to form an exterior cage that limited the expansion of the air-filled bladders. In so doing, the bladders could be made of a thinner polyurethane film (1 mil). Also, the adult size device has an inner chamber that is filled with saline to even out the compression. With the thinner, more compliant bladders, the saline chamber was eliminated in the pediatric version. Without the inner saline component, this prototype included a small fluid-filled bladder assembled between the epicardial interface of the device and the active bladders. A pressure sensor was advanced to sense the pressure in this custom pocket, providing a measure of instantaneous applied epicardial pressure. Although these refinements were developed for pediatric application, the reductions in bulk have utility for decreasing the invasiveness of the implantation for the adult design.

### Surgical Procedure

This study was conducted with the animal under general anesthesia, maintained by isoflurane in 100% oxygen. Buprenorphine 0.005 mg kg^− 1^ and xylazine 0.08 mg kg^− 1^ were administered intravenously for pre-anesthetic medications, followed by intravenous injections of ketamine 4 mg kg^− 1^ and diazepam 0.2 mg/kg for anesthetic induction. Lactated Ringer’s solution 5–20 mL kg^− 1^ h^− 1^ was used to maintain hydration and lidocaine 25–50 µg kg^− 1^ min^− 1^ was given intravenously to prevent arrhythmia during the procedure.

The physiologic parameters examined during this study included electrocardiogram (ECG), aortic pressure (AoP), central venous pressure (CVP), pulmonary artery pressure (PAP), left ventricular pressure (LVP), aortic flow (AoF), and pulmonary artery flow (PAF). The CVP and PAP were measured using a Swan-Ganz catheter placed via external jugular access, and AoP was acquired in the descending aorta using a fluid-filled pressure catheter via femoral artery access. The LVP was acquired using a Millar (Houston, Texas) VentriCath placed in the LV via carotid artery access. The AoF and PAF were acquired using Transonic (Ithaca, New York) COnfidence flow probes, placed around each artery via thoracotomy, taking care to maintain the ventricular pericardium. DCC system pressures and hemodynamic parameters were monitored using PowerLab data acquisition hardware and LabChart software (ADInstruments, Colorado Springs, Colorado). Fluoroscopy was used for visual guidance.

**Fig. 2:**
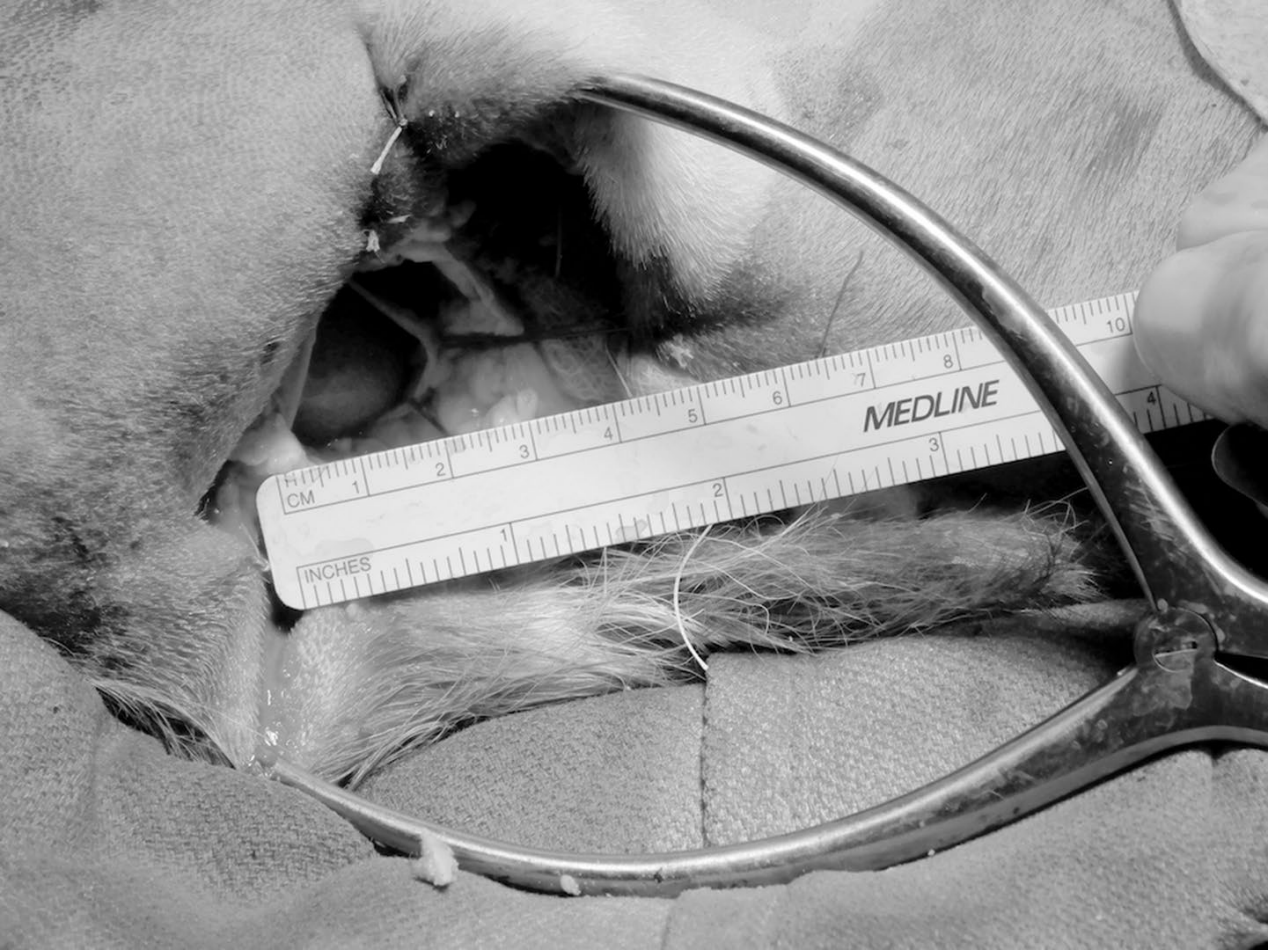
Photograph of minimally invasive surgical access for implantation of the pediatric DCC heart support implantable prototype device; the animal is shown in right lateral recumbency, with xyphoid access to the pericardial window at the apex of the beating heart

After placement of all hemodynamic data transducers, a 7 cm ventral incision was made below the sternum and a portion of the xyphoid process was removed. The apex of the beating heart was exposed with a 2.5 cm diameter incision in the pericardial sac. A pericardial cradle was performed using stay-sutures to stabilize the apical opening of the sac. The deployment tube with the preloaded implantable device prototype was positioned inside the pericardial window, and the prototype was deployed to reside within the pericardial sac around the heart. Figure 2 shows the minimally invasive surgical access used for device implant. The pericardial cradle sutures were released and the ventral incision was closed around the transcutaneous driveline.

### Data Sampling and Analysis

To achieve acute HF, a high-dose of esmolol was titrated by constant rate infusion of 600–800 𝜇g kg^−1^ min^−1^, targeting a 30–60% decline in cardiac output (CO) from pre-esmolol baseline. The animal was on device support during the HF status, other than to collect intermittent samples with device idle to acquire HF baseline.

Samples which did not meet study inclusion criteria (30–60% reduction in CO from pre-esmolol healthy baseline) were excluded from the analysis to allow comparison between relatively similar HF conditions. This was an exploratory study with several device settings tested, however only samples with similar implantable and drive system settings were included in this analysis. Five paired samples of device idle/device support were included to evaluate instantaneous effect of device activation with approximately 15 mmHg DCC pressure applied to the ventricles (n = 5 device idle, n = 5 device support); each sample was 20.0 s (23 ± 1 cardiac cycles), approximately 2 positive-pressure respirations. Results are presented as mean ± standard deviations within the averaged data pairs.

After completion of data collection, the esmolol was allowed to metabolize such that hemodynamics returned to healthy baseline conditions. To assess the device explantability from a beating heart (without cardiac support such as cardiopulmonary bypass, (CPB)), the xyphoid incision was partially reopened, and the implantable was retracted from the beating heart.

After explantation of the first prototype, a second prototype device was implanted (by same method) to evaluate proof-of-concept of DCC for emergency CPR. After implantation, cardiac arrest was induced with a 100 mg kg^− 1^ intravenous bolus of pentobarbital sodium. Upon confirming asystole, DCC was initiated and device support was triggered manually with a constant rate of 90 BPM. Hemodynamic data were sampled in blocks of 10.0 s and were sampled prior to asystole, during asystole induction, and three times during asystole after initiation of DCC (upon initiation, after 1 min, and after 2 min). Data values are 10.0 s averages. DCC was suspended to complete euthanasia protocol.

## Results

### Device Implantation

**Fig. 3:**
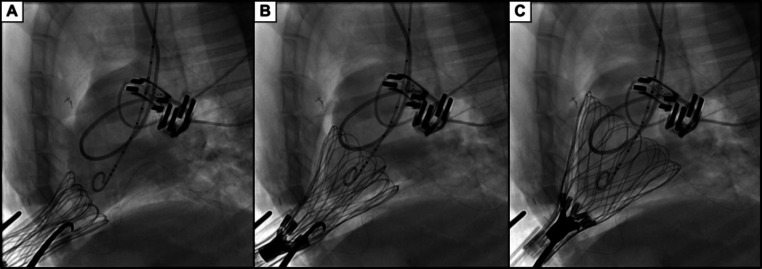
Fluoroscopic frames showing the pediatric heart support prototype self-expanding inside the pericardial sac; the super elastic nitinol frame guides the implantable out of the deployment tube (**A**) and into the pericardial sac (**B**) until surrounding the ventricles (**C**); also shown are the blood flow probes positioned around the pulmonary artery and the aorta, along with the Swan-Ganz catheter and the Millar Ventri-Cath left ventricular pressure-volume catheter with pigtail tip

**Fig. 4:**
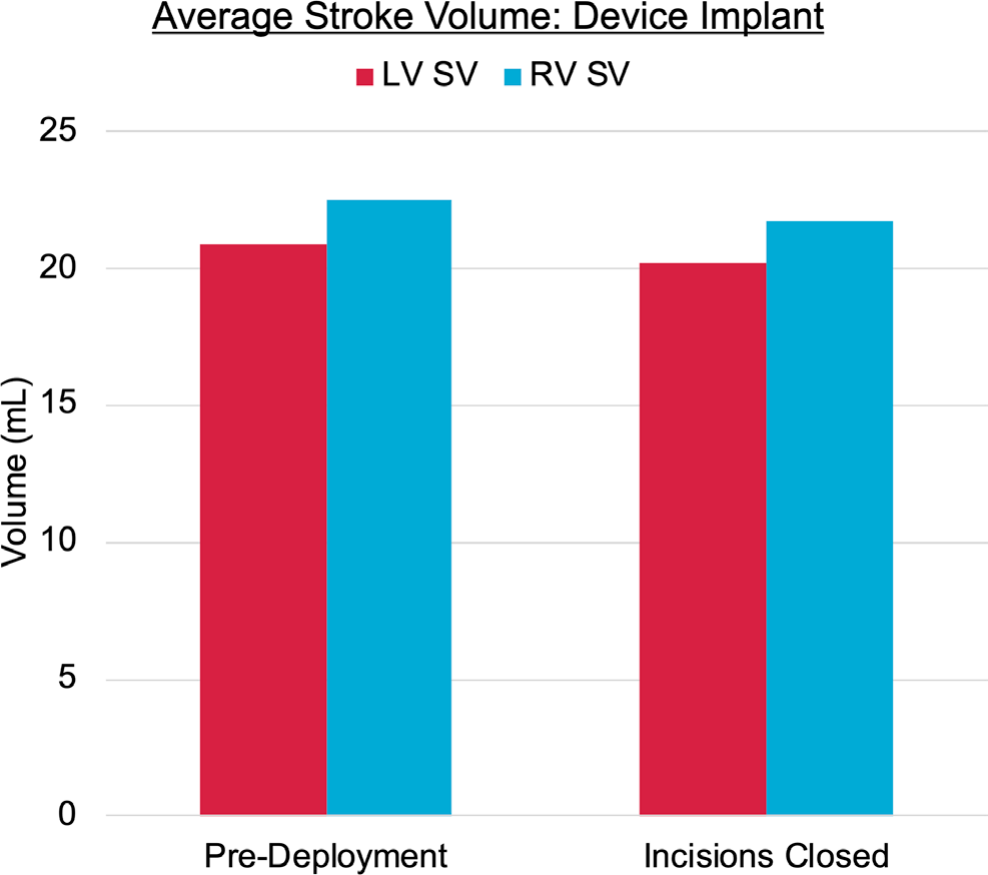
Bar chart showing the negligible change in stroke volume (SV) before and after deployment of the pediatric device; a negligible change represents successful deployment without impeding native heart function

The intrapericardial implantation of the device was successfully achieved via self-expansion around the epicardial surface of the ventricles. The procedure was performed on a beating heart without bypass. Appropriate device fit was verified by visual inspection of the fluoroscopic imaging (Fig. 3). This is supported by a negligible change in stroke volume (SV) between pre-deployment and after closing incisions (Fig. 4). Similarly, the changes in left- and right-heart filling pressures were negligible: left ventricular end-diastolic pressure (LVEDP) was 8 mmHg before deployment and 7 mmHg after closing; likewise, mean CVP was 8 mmHg before deployment and 7 mmHg after closing. This negligible change in filling pressures indicated no diastolic restriction with implant.

### Device Support during Heart Failure

After induction of HF using esmolol, the mean AoF/PAF declined by 46%/40% with a 34% decline in systolic blood pressure (SBP) and a 29% increase in mean CVP, establishing an acutely decompensated cardiac state < 10 min after initiating esmolol infusion. During HF, approximately 15 mmHg DCC pressure applied to the ventricles resulted in an immediate 40%/43% increase in AoF/PAF and a 41% increase in systolic LVP compared to HF without DCC support. Relative to baseline, DCC support during HF recovered 76% of baseline LV CO and 85% of baseline RV CO, and recovered 83% of baseline systolic LVP. The systolic arterial pressures were impacted more by DCC than diastolic and mean pressures; from HF, the SBP increased by 17% whereas mean aortic pressure (MAP) increased by 8% with DCC, and systolic PAP (PAPs) increased by 33%, with increase in mean PAP (PAPm) of 8% from HF. Relative to baseline, DCC assist during HF recovered 77% of the baseline SBP and 85% of baseline MAP.

**Fig. 5:**
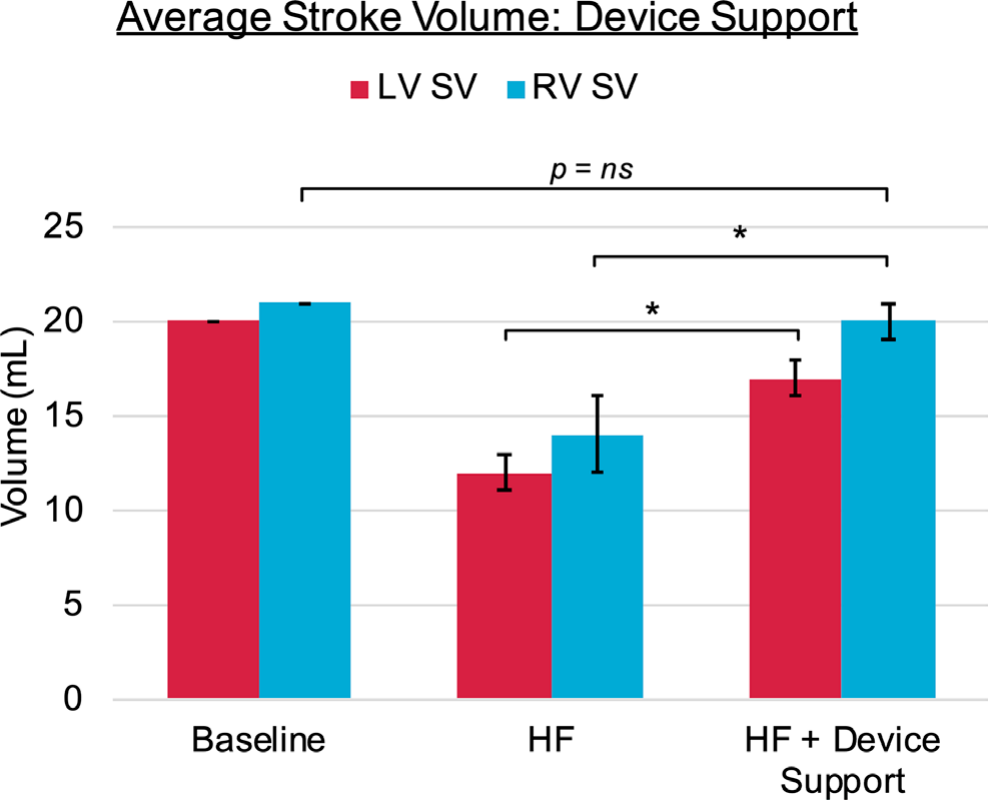
Graphical summary of the relative changes in left ventricular (LV) and right ventricular (RV) stroke volume (SV) from Baseline to esmolol-induced heart failure (HF), and from HF to HF with direct cardiac compression (DCC) device support (HF + Device Support); note that DCC resulted in a significant change in RVSV and LVSV (* p < 0.05), and the difference between the resulting supported RVSV and the Baseline RVSV was not statistically significant (p > 0.05)

When comparing the SV outcomes to the pre-HF healthy baseline, the device recovered 85%/95% of the pre-HF baseline LVSV/RVSV (SV_HF+DCC_/SV_pre−HF_); moreover, the RVSV with device support during HF did not differ significantly from the baseline RVSV (p > 0.05). The changes in SV with device support are illustrated in Fig. 5. All sampled hemodynamic parameters at baseline (prior to induction of HF), during HF, and the results with device support during HF are summarized in Table 1, with significant changes from HF indicated (p < 0.05).

**Table 1:** **Summary of Hemodynamics.** Summary of hemodynamic parameters at baseline pre-HF, during HF, and with device support during HF

	HR *(BPM)*	AoF *(L/min)*	PAF *(L/min)*	LV SV *(mL)*	RV SV *(mL)*	SBP *(mmHg)*	DBP *(mmHg)*	MAP *(mmHg)*	LVPs *(mmHg)*	LVP min *(mmHg)*	LVP EDP *(mmHg)*	PAPs *(mmHg)*	PAPd *(mmHg)*	PAPm *(mmHg)*	CVP *(mmHg)*
Baseline	72 ± 0	1.42 ± 0	1.51 ± 0	20 ± 0	21 ± 0	62 ± 0	39 ± 0	46 ± 0	54 ± 1	4 ± 0	7 ± 0	16 ± 0	10 ± 0	13 ± 0	7 ± 0
HF	65 ± 1	0.77 ± 0.11	0.9 ± 0.12	12 ± 1	14 ± 2	41 ± 2	32 ± 1	36 ± 1	32 ± 2	5 ± 0	9 ± 0	15 ± 0	10 ± 0	13 ± 0	9 ± 0
HF + Device Support	65 ± 1	*1.08 ± 0.07	*1.29 ± 0.08	*17 ± 1	*20 ± 1	*48 ± 2	*34 ± 2	*39 ± 2	*45 ± 1	*3 ± 0	9 ± 0	*20 ± 0	10 ± 0	14 ± 0	9 ± 0
∆	0 ± 1	0.31 ± 0.05	0.39 ± 0.07	5 ± 1	6 ± 1	7 ± 1	2 ± 1	3 ± 1	13 ± 1	-2 ± 0	0 ± 1	5 ± 1	0 ± 0	1 ± 0	0 ± 1

* indicates significant difference between HF and HF with device support (p < 0.05)

**Fig. 6:**
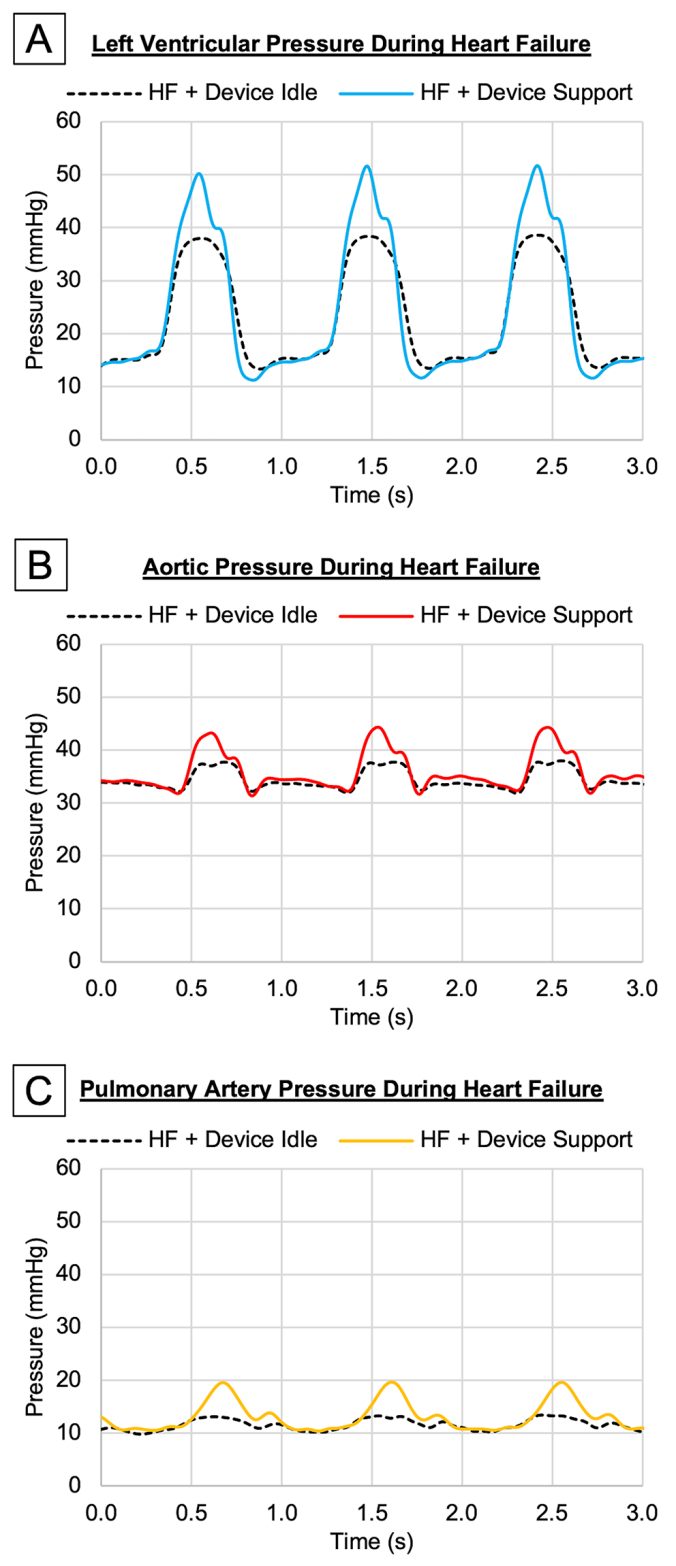
Representative waveforms to demonstrate the instantaneous hemodynamic pressure changes with device support; for each the left ventricular pressure [**A**], the aortic pressure [**B**], and the pulmonary artery pressure [**C**], the heart failure state while the device is idle (HF + Device Idle) is overlaied with the resulting change in hemodynamic pressure with device support (HF + Device Support)

**Fig. 7:**
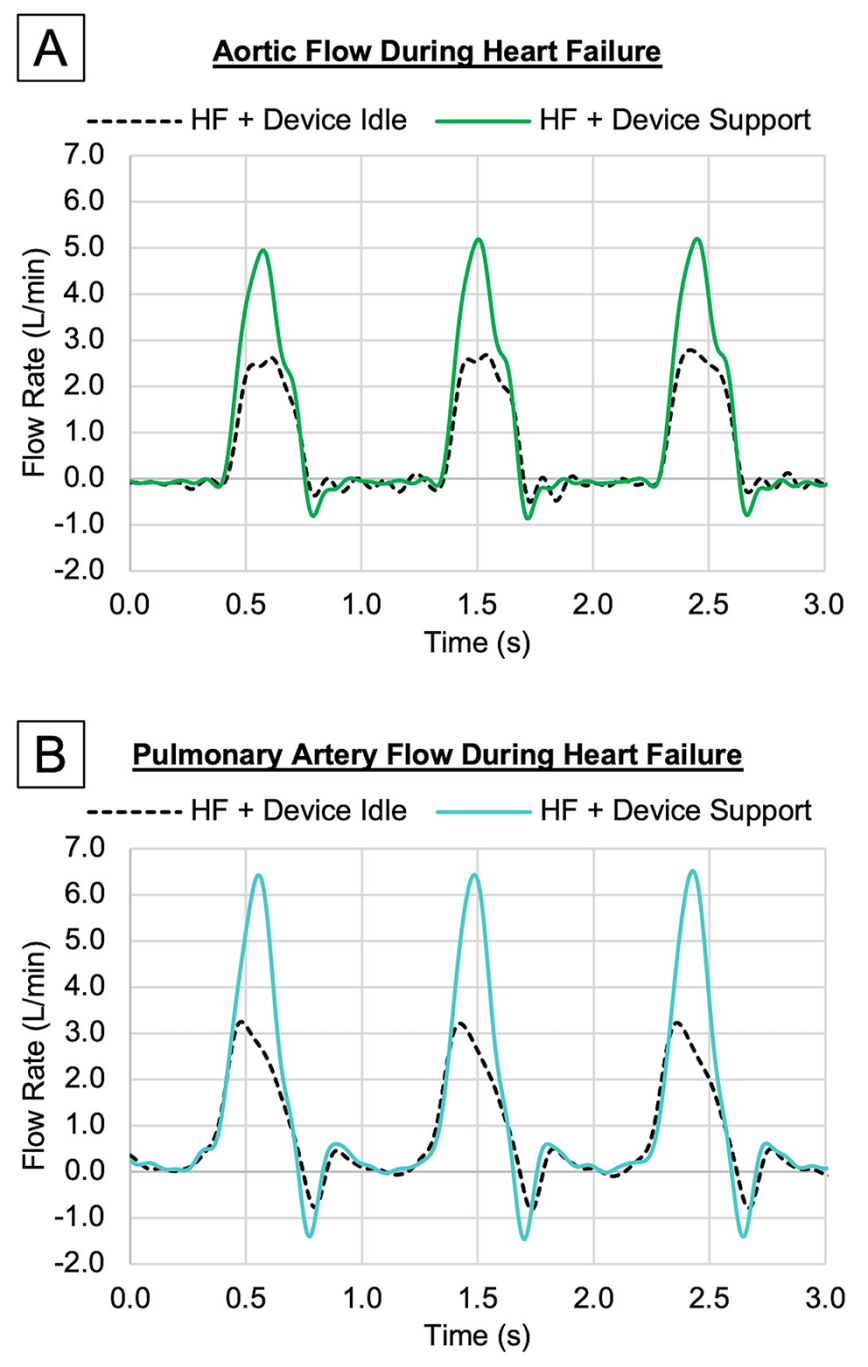
Representative waveforms to demonstrate the instantaneous hemodynamic flow changes with device support observed during this study; for each the aortic flow [**A**] and the pulmonary artery flow [**B**], the instantaneous blood flow during heart failure state while the device is idle (HF + Device Idle) is overlaied with the resulting instantaneous blood flow rate with device support (HF + Device Support)

Waveforms in Figs. 6 and 7 illustrate representative samples of HF hemodynamics prior to support with the device idle (dotted black lines) and during device support (solid-colored lines). The hemodynamic waveforms in Fig. 6 reflect the instantaneous change in inflation and deflation rate of the active assist bladders. These samples demonstrate that the pulsatile hemodynamics of native heart function were maintained during device operation. Additionally, while the improvement is most noticeable during the systolic phase, the reduced early diastolic filling pressure in LVP (Fig. 6A) indicates improved rapid filling, or diastolic assistance.

### Device Removal

After hemodynamics returned to baseline with discontinuation of esmolol, the xyphoid incision was partially reopened and the implantable was retracted through the incision by gently pulling on the driveline hub. The superelastic Nitinol frame collapsed easily and atraumatically when retracted via the apical opening in the pericardium and the sub-sternal skin incision. This was performed on a beating heart without the need for cardiopulmonary bypass. No systemic anticoagulation measures were used during the procedure. Supplemental video S1 has been included to demonstrate the straightforward minimally invasive removal of the prototype from the beating heart.

### Asystolic Cardiac Arrest

**Fig. 8:**
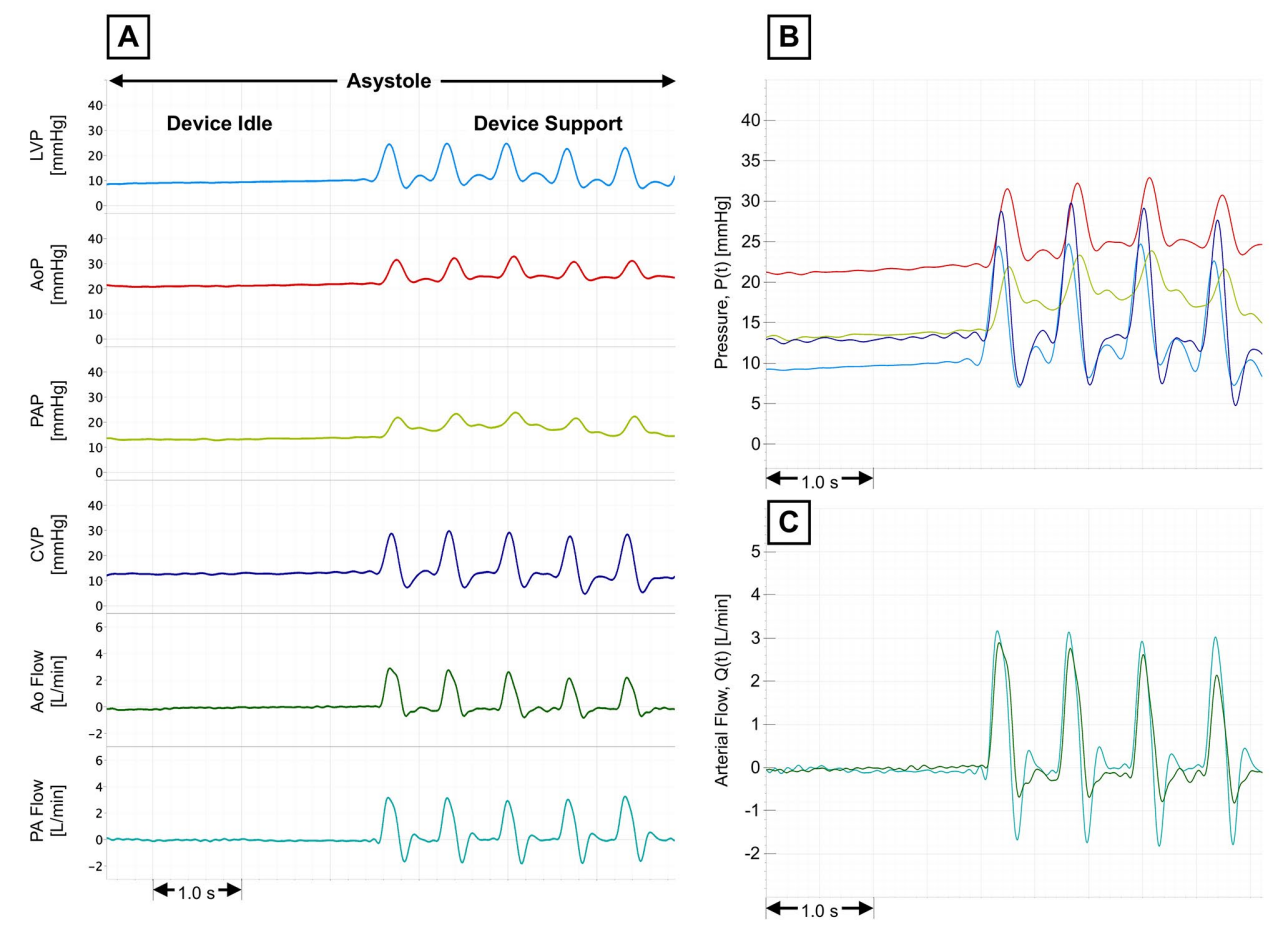
Hemodynamic waveforms sampled during DCC device support on an asystolic arrested heart; **A**: waveforms sampled at initiation of 90 BPM DCC during asystole, including 1: left ventricular pressure (LVP, light blue), 2: aortic pressure (AoP, red), 3: pulmonary artery pressure (PAP, yellow), 4: central venous pressure (CVP, dark blue), 5: aortic flow (Ao, green), and 6: pulmonary artery flow (PA, teal); **B**: waveform overlay of all pressures – note that CVP (dark blue) is synchronized and comparable to LVP (light blue), likely indicating some degree of right ventricular papillary insufficiency and right atrial regurgitation during systolic ventricular compression; **C**: arterial flow waveform overlay at initiation of DCC during asystole

Data were briefly collected to evaluate proof-of-concept for DCC CPR on an arrested heart. Quantitative results are summarized in Table 2. At most, DCC sustained between 73% and 79% of the systemic blood pressures measured pre-cardiac arrest; pulmonary pressures during DCC CPR exceeded the pulmonary pressure seen prior to cardiac arrest. After 2 min, the systolic pulmonary and systemic blood pressures were comparable, possibly indicating a blood pressure equilibrium with prolonged compression of both ventricles with the same pressure. At most, DCC during cardiac arrest raised CO to 44% of the pre-arrest CO. Of note, the maximum CO seen with DCC during cardiac arrest was similar to the absolute change in CO with DCC during HF (beating heart). At 1 min of DCC during asystole, the AoF was 0.48 L/min (at 90 DCC/min, SV_DCC_ = 5 mL), compared to a mean absolute increase 𝛥AoF of 0.31 ± 0.05 L/min at 65 BPM (𝛥SV = 5 mL) with device support on the beating heart during HF. Hemodynamic waveforms sampled at DCC initiation during asystole are shown in Fig. 8. Of note, the CVP waveform is noticeably elevated during systole; CVP was synchronized and comparable in magnitude to ventricular compression. This unusually high pressure in the right atrium during systole may indicate some degree of right ventricular papillary insufficiency and right atrial regurgitation during ventricular compression on the arrested heart.

**Table 2:** **Cardiac Arrest Hemodynamics.** Summary of hemodynamic parameters during assessment of DCC for CPR on an asystolic arrested heart

Avg. Hemodynamics	Prior to Cardiac Arrest	Asystolic Cardiac Arrest	DCC Initiation	DCC 1 min	DCC 2 min
Systemic Pressures (mmHg)					
DBP	38	21	24	24	30
SBP	52	24	32	34	38
MAP	45	22	27	28	33
Pulmonary Pressures (mmHg)					
PAPd	11	13	15	16	22
PAPs	18	15	23	24	31
PAPm	15	13	18	19	25
Cardiac Output (L/min)					
AoF	1.10	-0.06	0.28	0.48	0.23
PAF	1.24	-0.03	0.43	0.41	0.25

## Discussion

Current pediatric MCS options are prone to complications, with several of these complications arising from the device-blood interface and disruption of physiologic hemodynamics; thus, DCC provides an opportunity to mitigate risks associated with MCS. Previous attempts with DCC for pediatric HF have included the PediBooster and the use of cardiac artificial rubber muscles by Saito et al. [15, 16]. The PediBooster featured two polyurethane balloons for co-pulsation compression of each ventricle with systolic inflation of the balloons. The design by Saito et al. featured two circumferential rubber artificial muscles with a maximum permissible contraction of 25% of the normal circumference during systolic inflation. While both resulted in promising preclinical proof-of-concept for improved CO support without blood contact in an animal model of HF, both designs resulted in increased ventricular filling pressures during device activation. This may be resolved in future studies by the authors of each design. To our knowledge, there has been no prior construction of a pediatric DCC device which provides support without significant diastolic restriction or damage to heart tissue and does not require an adhesive agent to couple the DCC implantable to the heart. This proof-of-concept study demonstrates construction of a DCC device on a pediatric scale without previously encountered limitations.

### Pediatric Device Development

Towards prototyping a DCC device sized for a pediatric heart, high-fidelity 3D imaging modalities such as cardiac-gated CT or MRI would likely produce the most accurate dimensions, yet the fluoroscopic C-arm method for preclinical research pre-study imaging had its advantages. The pre-study images in this study were captured with the animal conscious and standing, whereas CT and MRI both would require the animal to be anesthetized and recumbent for image acquisition. General anesthesia of large animals adds risk and expense to a research study design, and thus was avoided in the present study. Transthoracic echocardiography (echo) is relatively low-cost in comparison to CT or MRI and can potentially be used on a conscious animal. However, the echo acoustic windows in the ruminant anatomy limit full-visibility of the exterior boundaries of the heart because the heart is positioned more medially and the apex more ventrally tilted than in humans. The central axis of the goat heart is approximately in the middle of the chest; therefore, radiopaque scales positioned on the left and right sides of the animal could be averaged together to approximate the scale in the middle of the chest (at the depth of the heart) during sagittal imaging. Finally, fluoroscopic cines of multiple consecutive cardiac cycles allowed us to capture a frame during end-diastole that avoids end-expiration (when the diaphragm would be expanded towards heart). The maximum axial height and posterior-anterior dimensions were obtained and revolved to form a 3D approximation of the heart, and the intraoperative fluoroscopic imaging and hemodynamic parameters sampled during the device implant procedure verified that the prototypes were an appropriate size for this preclinical model of a pediatric heart.

### Non-Blood Contacting

As current MCS technology is scaled in size to fit pediatric patients, the ratio of the foreign-body surface areas contacting the blood (of the cannulas and the pump itself) relative to the volume of the blood increases. Moreover, from a fluid mechanics standpoint, pediatric patients have a lower blood flow velocity and smaller luminal diameter than adult patients. Thus, the propensity for thrombosis during MCS is increased both in vivo and within the smaller cannula sizes used for pediatric ECMO as compared to the adult population [19]. Furthermore, anticoagulation management is more complex in pediatric patients due to age-related changes in hemostasis as well as coagulation disorders associated with CHD [4, 20]. The increased exposure to allogeneic blood products during blood-contacting MCS in the context of the immunologic, cardiac, and renal immaturity of childhood, poses a greater risk of transfusion complications such as transfusion-related immunomodulation, hypocalcemia, hyperkalemia, and acidosis [21, 22]. These complications have remained unchanged because, clinically, there is no better option than ECMO.

The results of this study show that temporary MCS may be able to be achieved without blood contact. The device recovered 76% of LV CO and 85% of RV CO during in vivo testing in the preclinical HF model. Relative to the results for the adult-sized CorInnova DCC device, the pediatric results may be more clinically important because the risk of thromboembolisms, hemorrhagic events, and infections resulting from device-blood interfacing in children is several-fold greater than the risk observed in adults with blood-contacting MCS [19, 23, 24]. The disproportionate risk of adverse events incurred by pediatric patients treated with MCS merits innovative solutions, such as DCC.

### Support of Physiologic Cardiovascular Mechanics

Biventricular heart support with the DCC prototype recovered up to 95% of baseline SV without significant alteration of the LVSV/RVSV ratio typically observed with ECMO and VAD support [25, 26]. Clinically, prolonged single-ventricle MCS often results in overloading and concomitant failure of the opposite ventricle. Specific to PC-ECMO, conventional VA-ECMO cannulation often results in high afterload on the LV and low preload on the RV. This hemodynamic imbalance can be addressed by venting through an atrial shunt or additional cannulation for drainage, but adds to the complexity of the MCS. These perturbations of preload and afterload limit the opportunity for heart recovery and device removal. The maintenance of normal cardiac geometry and biomechanics has been shown to facilitate myocardial repair and functional recovery [27]. Thus, the ability to improve CO without significant perturbations of LVSV/RVSV ratio could represent the ability of pediatric DCC to improve the success of bridge-to-recovery MCS and should be investigated further.

As shown in Figs. 6 and 7, and Fig. 8, DCC produced pulsatile hemodynamics during support. The use of pulsatile perfusion in pediatric CPB has been seen to positively affect the post-operative recovery process [28]. Pulsatile MCS has been shown to improve systemic microcirculation [29], decrease levels of pro-inflammatory cytokines such as IL-1β and TNF-ɑ [30], and decrease the magnitude of cerebrovascular dysregulation when compared to non-pulsatile MCS [31]. While the physiologic evidence supports the benefits of pulsatile flow, designing and producing pulsatile blood pumps has proved challenging from an engineering standpoint and successful clinical application has been limited. Thus, standard-of-care ECMO is innately non-pulsatile. The ability to maintain physiologic pulsatile flow could represent a strategy to mitigate the mortality and morbidity resulting from the suboptimal microcirculatory perfusion, cerebrovascular dysregulation, inflammation, and pathologic remodeling observed in non-pulsatile MCS.

Implantation of the extracardiac prototype did not impact the normal physiologic diastolic filling pressures. Additionally, during device support, the device did not demonstrate diastolic restriction. The LV hemodynamics remained relatively unchanged during end-diastole, and were actually improved during early diastole (Fig. 6). This potentially indicated assistance with rapid filling and improved diastolic filling. This is significant progress as prior pediatric DCC devices had been limited by the issue of diastolic restriction in vivo [15, 16]. In addition to augmenting CO, unrestricted diastolic motion maintains normal cardiac geometries which could facilitate myocardial recovery.

### Atraumatic Device Implant, Activation, and Removal

Within the acute study window, the DCC deployment, ventricular support, and removal did not result in any gross trauma to the myocardium, pericardium, or epicardium. Previous pediatric DCC devices have demonstrated gross contusions to the myocardium as a result of the compressive force [16]. To our knowledge, there has been no prior pediatric DCC device able to improve CO in this range without gross damage to heart tissue. These improvements in DCC safety (no diastolic restriction, atraumatic, and minimally invasive explantation) are likely attributable to the design, wherein a soft, collapsible device was sought that mimicked the end-diastolic shape when deflated and the end-systolic shape when inflated. With the device shape similar to that of the heart when actuating, there is less potential for interference with native heart function. Similar atraumatic results are predicted for future, longer term studies.

The minimally invasive removal technique shown in supplemental video S1 demonstrates improvement over prior pediatric DCC device removal techniques as well as the potential to avoid invasive surgical procedures in medically fragile patients [15, 16]. If an ECMO weaning trial is successful, final weaning is performed in an operating suite immediately followed by decannulation [32]. In pediatric patients receiving PC-ECMO, central cannulation is most common [33, 34], and it requires open-chest surgery to decannulate, resulting in further risk to the patient [35]. Low-flow states observed in the ECMO circuit during weaning trials further increase the risk of clot formation within the cannulas and further perturb hemostatic parameters [36, 37]. Additionally, blood-contacting MCS cannot be fully paused to confidently determine if the patient is ready for ECMO removal. The results here show that DCC pressure can be reduced and paused completely: the device is non-obligatory and can be turned off to evaluate the status of native heart function and overall readiness for device removal. The minimally-invasive surgical removal of the intrapericardial DCC prototype was achieved on a beating heart and did not require cardiopulmonary bypass.

### Asystole Support

To evaluate the potential for this DCC prototype for CPR, DCC support was performed during a cardiac asystolic state. After asystole was confirmed, the device was activated at a rate of 90 BPM. The device achieved recovery of 21–44% of pre-arrest CO as shown in Table 2; for comparison, one study found that external manual compressions performed during CPR recover 20–30% of pre-arrest CO [38].

The ability to incorporate DCC into CPR clinical management algorithms could yield improvement in clinical outcomes. Studies have demonstrated greater success with circumferential external cardiac compression than traditional top-down manual external compressions [39, 40]. Furthermore, direct cardiac massage achieves greater increases in aortic and coronary pressures than external cardiac compressions, both of which are correlated with a greater likelihood of achieving return of spontaneous circulation [41]. With regards to PC-ECMO, central cannulation complicates the success of chest compressions, and the ability of DCC to provide circulatory support during asystole could provide an alternative to ECMO in CPR [42].

### Prototype Durability

Short-term use is anticipated to be approximately 8–10 days based on the mean duration of ECMO support of 8.4 days [43]. This would indicate benchtop durability should exceed 20 days to achieve a 2x safety factor. Benchtop testing on a heart shaped, thin-walled structure (connected to a 30mmHg water column to counteract the 30mmHg of device pressure), demonstrated feasible durability of prototypes for > 30 days. Durability cannot be fully established until manufacturing methods are defined and implemented.

### Limitations

This study has several limitations inherent to proof-of-concept work. The data presented was obtained using the best of three different prototype designs and shows what is possible in a single animal. The clinical utility remains unknown. Furthermore, it remains unknown if the prototype can reliably reproduce similar results in multiple animals. The study is also limited by the preclinical model which approximates, but does not precisely mimic, pediatric HF. While esmolol-induced HF is a more appropriate model of HF than ischemia for pediatric applications [44], esmolol results in significant vasodilation and a degree of systemic interference in hormonal control of circulation that is not present in pediatric HF. Finally, the procedures required to place the instrumentation for this experiment in the animal model (i.e. arterial flow probes, LV pressure catheter) added more surgical time and complexity than what is to be expected in future clinical trials of this DCC technology.

### Concluding Remarks

This study represents a first step towards development of a novel solution in the pediatric space where the need is great and yet innovation is lacking. Proof-of-concept was successfully demonstrated for minimally invasive in vivo surgical implantation of the pediatric DCC prototype on a beating heart, cardiac support during HF, feasibility of minimally invasive removal from a beating heart, and emergency CPR during asystolic cardiac arrest. Future directions to address the limitations of this study include pediatric HF model refinement by using juvenile animal models which more closely resemble pediatric cardiovascular physiology [44]. Additionally, given the critical need for better MCS therapies for single-ventricle patients, preclinical investigation in a failing Fontan model may be explored.

### Electronic Supplementary Material

Below is the link to the electronic supplementary material.

Supplementary Material 1

Supplementary Material 2

## Table of Acronyms

AoF: Aortic Flow
AoP: Aortic Pressure
CAD: Computer-Aided Design
CHD: Congenital Heart Disease
CO: Cardiac Output
CPR: Cardiopulmonary Resuscitation
CPB: Cardiopulmonary Bypass
CT: Computed Tomography
CVP: Central Venous Pressure
DCC: Direct Cardiac Compression
ECG: Electrocardiogram
ECMO: Extracorporeal Membrane Oxygenation
HF: Heart Failure
LV: Left Ventricle
LVEDP: Left Ventricular End-Diastolic Pressure
LVP: Left Ventricular Pressure
MAP: Mean Aortic Pressure
MCS: Mechanical Circulatory Support
MRI: Magnetic Resonance Imaging
OHT: Orthotopic Heart Transplantation
PAF: Pulmonary Artery Flow
PAP: Pulmonary Artery Pressure
PC: Post-Cardiotomy
RV: Right Ventricle
SBP: Systolic Blood Pressure
SV: Stroke Volume
VAD: Ventricular Assist Device
